# Genomic analysis of respiratory syncytial virus infections in households and utility in inferring who infects the infant

**DOI:** 10.1038/s41598-019-46509-w

**Published:** 2019-07-11

**Authors:** Charles N. Agoti, My V. T. Phan, Patrick K. Munywoki, George Githinji, Graham F. Medley, Patricia A. Cane, Paul Kellam, Matthew Cotten, D. James Nokes

**Affiliations:** 1 Kenya Medical Research Institute (KEMRI)—Wellcome Trust Research Programme, Epidemiology and Demography Department, Kilifi, Kenya; 2 Pwani University, School of Health and Human Sciences, Kilifi, Kenya; 3 Wellcome Trust Sanger Institute, Cambridge, United Kingdom; 4 London School of Hygiene and Tropical Medicine (LSHTM), Department of Global Health and Development and Centre for Mathematical Modeling of Infectious Disease, London, United Kingdom; 5 Public Health England, Porton Down, Salisbury, United Kingdom; 6 Imperial College London, Department of Infection, London, United Kingdom; 7 University of Warwick, School of Life Sciences and Zeeman Institute, Coventry, United Kingdom; 8 Present Address: Erasmus Medical Center, Department of Viroscience, Rotterdam, The Netherlands; 9 Present Address: Center for Disease Control and Prevention, Division of Global Health Protection, Nairobi, Kenya; 10 Present Address: MRC/UVRI & LSHTM Uganda Research Unit, Entebbe, Uganda; 11 Present Address: MRC-University of Glasgow Centre for Virus Research, Glasgow, UK

**Keywords:** Phylogenetics, Viral epidemiology, Viral evolution, Viral transmission

## Abstract

Infants (under 1-year-old) are at most risk of life threatening respiratory syncytial virus (RSV) disease. RSV epidemiological data alone has been insufficient in defining who acquires infection from whom (WAIFW) within households. We investigated RSV genomic variation within and between infected individuals and assessed its potential utility in tracking transmission in households. Over an entire single RSV season in coastal Kenya, nasal swabs were collected from members of 20 households every 3–4 days regardless of symptom status and screened for RSV nucleic acid. Next generation sequencing was used to generate >90% RSV full-length genomes for 51.1% of positive samples (191/374). Single nucleotide polymorphisms (SNPs) observed during household infection outbreaks ranged from 0–21 (median: 3) while SNPs observed during single-host infection episodes ranged from 0–17 (median: 1). Using the viral genomic data alone there was insufficient resolution to fully reconstruct within-household transmission chains. For households with clear index cases, the most likely source of infant infection was via a toddler (aged 1 to <3 years-old) or school-aged (aged 6 to <12 years-old) co-occupant. However, for best resolution of WAIFW within households, we suggest an integrated analysis of RSV genomic and epidemiological data.

## Introduction

Respiratory syncytial virus (RSV) is a leading viral cause of bronchiolitis and pneumonia during infancy^1^. Global estimates in 2015 indicated that RSV causes ~33.1 million episodes of acute lower respiratory tract illness annually, ~3.2 million of which lead to hospital admissions and ~60,000 deaths in hospitalized children aged under 5 years^1^. Despite this burden, our understanding of RSV transmission patterns during epidemics, including who infects the vulnerable infant populations remains incomplete^2^. Defining the patterns of RSV transmission during epidemics, and specifically Who Acquires Infection From Whom (WAIFW) has the potential to inform control strategies^3,4^.

RSV transmission occurs during contact with an infectious person or contaminated environmental surfaces^5^. Households are considered an important setting for RSV spread due to likely close person-to-person contacts^6,7^. A family study in the United States in the 1970s showed that up to 46% of family members and 62% of infants in the household become infected once the virus is introduced into a household^8^. Since this study, important advances have been made in diagnostic sensitivity and characterisation of infection sources for household cases, in contact mapping tools and in statistical methods to infer epidemiologically linked case pairs^9,10^. Furthermore, household demographic characteristics may differ between developed and developing settings^11^.

Currently, there is no licensed RSV vaccine, although there are 19 vaccine, prophylactic or monoclonal antibody candidate products in clinical trials^12,13^. Impediments to RSV vaccine discovery have been the need to immunize in the first weeks of life when infant immune responses are still sub-optimal and enhanced disease observed during a formalin inactivated vaccine trial in the late 1960s^14^. Live attenuated vaccines given intranasally, have proved difficult to sufficiently attenuate to limit upper airway congestion during vaccination, while still maintaining immunogenicity^15,16^. As a result, alternative approaches are being considered including boosting infant antibody levels through maternal sub-unit vaccine immunization, pre-season delivery of high titre extended half-life immunoglobulin, reducing virus circulation in the community by vaccination of older babies and children or by cocoon vaccination to interrupt chains of transmission leading to infant infection^4,17,18^. To advance the cocoon vaccination strategy, a better understanding of RSV transmission in household settings where most transmissions appear to occur is required^18^.

Currently, little is known about the sequence change patterns during individual RSV infection episodes, or during intra-household and inter-household transmission events^10^. It is unclear if the pace of RSV genomic change is sufficient to allow tracking of transmission during epidemics. We have previously shown that partial RSV nucleotide sequences from the highly variable attachment (G) encoding gene (~900 nt) provide insufficient discriminatory power to delineate RSV transmission chains^19–21^. However, our initial analysis of RSVA full genome sequences (~15,200 nt) showed significant promise in providing phylogenetic resolution of viruses circulating in different households^10^ and similar application of these methods have been shown for norovirus^22^, foot and mouth disease virus^23^, influenza A virus^24^, MERS-CoV^25^, and Ebola virus^26,27^. In this study, we aimed to determine if RSV transmission in households is trackable using viral genomic data and if it is possible to identify who is the likely infector of the under 1-year-old infant.

## Materials and Methods

### Study location, design and samples

The study was undertaken within Kilifi County, which is located in coastal Kenya. A detailed description of the study location and study design was provided elsewhere^28^. Briefly, 47 households scattered across an area of approximately 21 km^2^ were followed up over a 6-month period beginning December 2009 and ending June 2010 coinciding with the RSV peak activity months in the area^29^. Households were defined as a group of people sharing a compound and eating from the same kitchen^20^. The selected households (abbreviated HHs) were given designated identifiers from 1 to 57 (HH01 to HH57). Twice weekly throughout the study period, a nasopharyngeal-flocked swab (NPS) was obtained from each member regardless of symptom status. The NPS samples were screened for RSV using a multiplex real-time RT-PCR method which subtyped RSV positives into RSVA and RSVB^30^. For whole genome sequencing (WGS), we targeted 20 select households that documented RSV infection of ≥2 members. A geographical map showing the distribution of the study households is provided in the Additional File: Fig. S1.

### RNA extraction, amplification and whole nucleotide sequencing

Viral RNAs from the positive samples of selected households were obtained using the QIAamp viral RNA extraction Kit (QIAGEN, Hilden, Germany) following the manufacturer’s instructions. Complementary DNA (cDNA) synthesis and RSV whole genome amplification was achieved using a six-overlapping PCR fragments strategy (each ~2.5 kb) as previously described^21^. Sequencing libraries were prepared using Nextera DNA Library Prep kits and nucleotide sequencing performed using Illumina MiSeq platform multiplexing 15–20 samples per run to generate approximately 1 million paired-end reads (150 bp × 2) per sample^21^.

### Whole genome sequence assembly and multiple sequence alignments

The short reads from the MiSeq instrument were de-multiplexed, quality checked (median read Phred score of ≥35) and trimmed using QUASR v6.08^31^. Reads passing quality checks were *de novo* assembled into longer contigs using the SPAdes v3.5.0^32^. RSV contigs were identified by matching to a database of RSV sequences using USEARCH program^33^, examined for completeness of the expected open reading frames (ORF) using Geneious v8.1.6 (https://www.geneious.com) and, where necessary, partial contigs were further combined to longer ones using Sequencher v5.0.1^34^. These were subsequently checked presence of intact ORFs, sorted by household, re-aligned and positions of nucleotide variation double-checked if these were supported by majority of the raw reads associated with that sample^10^. Multiple sequence alignments were prepared in MAFFT v7.220^35^.

### Phylogenetic analysis

Sequence phylogenies were inferred using Maximum Likelihood (ML) methods in MEGA7^36^ and RAxML v8.2.12^37^. The best-fitting models of nucleotide substitution for each alignment were in IQ-TREE v1.4.3^38^. Best tree search was performed by Nearest Neighbor Interchange (NNI). Branch support was evaluated by bootstrapping with 1,000 replicates. Pairwise genetic distances were calculated in pairsnp 0.0.6^39^. The phylogenetic relatedness of the household RSVA and RSVB genomes was assessed at three levels; (i) in combination with global sequences deposited in GenBank (RSVA, n = 657 collected between 1977–2015 while RSVB, n = 416 collected between 1978–2016), (ii) among the households viruses alone and (iii) among viruses collected from same households only. The potential transmission networks within and between households for each group were inferred in PopART package v1.7.2 using median joining tree (MJT) method with an epsilon of zero^40^. Evolutionary analyses were determined in maximum-likelihood-based TreeTime program^41^. Phylogenetic trees were visualized and annotated in FigTree v1.4.4 (http://tree.bio.ed.ac.uk/software/figtree/).

### Identifying who infected the household infant (s)

Infants were defined as the participants aged <1-year-old during the study^20^. We grouped the other participants into 5 age-groups: (i) toddlers (1 to <3 years), (ii) pre-schoolers (3 to <6 years), (iii) school-aged (6 to <12 years), (iv) adolescents (12 to <18 years) and adults (>18 years). We attempted to identify who among these other age-groups were the most likely infectors of the infants by examining the relatedness of the virus genome(s) obtained from the infant to all the viral genomes obtained from the other members in the same household. Information on the dates of the sampling of the sequenced samples was taken into account to position the infant in the transmission network/chain.

### Sequence nomenclature and accession numbers

The sequence nomenclature of the household samples has four digits that include the household identifier (first two digits) and subject identifier (the last two digits). All the new 112 full or partial RSVB genome sequences from this study were deposited in GenBank under the accession numbers MH594350 – MH594461. The RSVA genomes are deposited in GenBank under accession numbers KX510136-KX510266.

### Ethical approval

The samples were collected after obtaining informed written consent from each participant if aged ≥18 years or through a guardian or parent if aged <18 years. In addition, children aged above 5 years were asked for assent. The study protocol approved by both the Scientific and Ethics Review Unit (SERU) of the Kenya Medical Research Institute (KEMRI), Nairobi, and Coventry Research Ethics Committee of UK^20^. All study procedures were performed in accordance with the approved protocol guidelines and in compliance with the relevant regulations.

## Results

### RSV infections and whole genome sequencing

We targeted 20 households with a total of 226 occupants (range 4–37 persons per household) for WGS. Details of the demographic characteristics of the analysed households, total specimens collected, diagnostic results, genome sequencing success and the observed phylogenetic clades (defined later) are summarized in Table 1. Over the six-month period (December 2009 – June 2010), a total of 7,695 nasopharyngeal-flocked swabs (NPS) were collected from the 20 HHs, 415 (5.4%) of which were determined to be RSV real-time RT-PCR positive (cycle threshold (Ct) value of <35.0; 189 RSVA, 214 RSVB and 12 RSVA/B co-infections) these originating from 130 participants. Of the 415 positive specimens, 374 (90.1%) samples were processed for WGS^21^ with successful amplification and assembly of RSV contigs of >1000 nucleotides length in 246 samples (65.7%). Of these 191 samples (51.1%) yielded contigs >14000 nucleotides (nt) (103 RSVA and 88 RSVB i.e. >90% of RSV full-length genomes) hereafter referred to as genomes. In eight and 14 HHs, two or more RSVA or RSVB genomes were recovered, respectively, allowing our investigation into within-household RSV transmission and variation, Table 1. Genome sequencing success negatively correlated with increasing diagnostic RT-PCR Ct value. These results, together with details on the metadata of the sequenced RSVB viruses, GenBank and Sequence Read Archive accession numbers and assembly metrics are provided in the Additional File and Supplementary Dataset, respectively.

**Table 1:** Demographic details of the 20 households, number of positive samples, number of sequenced samples and clade assignment.

HH^€^ ID	HH05	HH06	HH12	HH14	HH17	HH18	HH19	HH25	HH26	HH29	HH31	HH35	HH38	HH40	HH41	HH42	HH45	HH49	HH51	HH57	Total
HH size	37	6	20	6	5	8	14	4	5	7	11	8	23	5	8	6	10	12	15	16	**226**
Females	24	6	10	2	3	4	8	1	4	3	8	5	10	2	5	5	7	5	11	7	**130**
In school	9	3	6	3	2	4	7	0	3	3	3	4	10	2	5	3	8	3	7	8	**93**
NPS^¥^ collected	1050	229	503	262	208	333	524	166	232	296	216	326	875	226	360	217	389	372	524	387	**7695**
RSV + ve	71	18	18^£^	18	11	11	31^£^	10	14^£^	25^£^	11	14	49	12	13	11	23^£^	16	19	20	**415**
RSVA + ve	70	2	1	18	0	0	1	0	9	25	11	0	24	12	0	0	6	2	2	18	**201**
RSVB + ve	1	16	18	0	11	11	31	10	7	6	0	14	25	0	13	11	19	14	17	2	**226**
Genomes sequenced	24	9	5	12	3	4	16	8	11	12	5	4	38	10	5	4	4	2	6	9	**191**
RSVA/I	24	1	—	12	—	—	—	—	9	12	5	—	22	10	—	—	—	—	—	8	**103**
RSVB/I	—	—	—	—	3		16	—	—	—	—	4	16	—	—	1	—	—	—	—	**40**
RSVB/II	—	8	5		—	4	—	—	—	—	—	—	—	—	1	—	—	2	—	—	**20**
RSVB/III	—	—	—	—	—	—	—	—	—	—	—	—	—	—	—	3	—	—	6	—	**9**
RSVB/IV	—	—	—	—	—	—	—	8	2	—	—	—	—	—	4	—	4	—	—	—	**18**
RSVB/V	—	—	—	—	—	—	—	—	—	—	—	—	—	—	—	—	—	—	—	1	**1**

^**€**^HH = household; ID = Identity; ^¥^NPS = nasopharyngeal-flocked swab; ^£^some member showed RSVA and B coinfection.

### Diversity of the viruses isolated in the study

**Figure 1:**
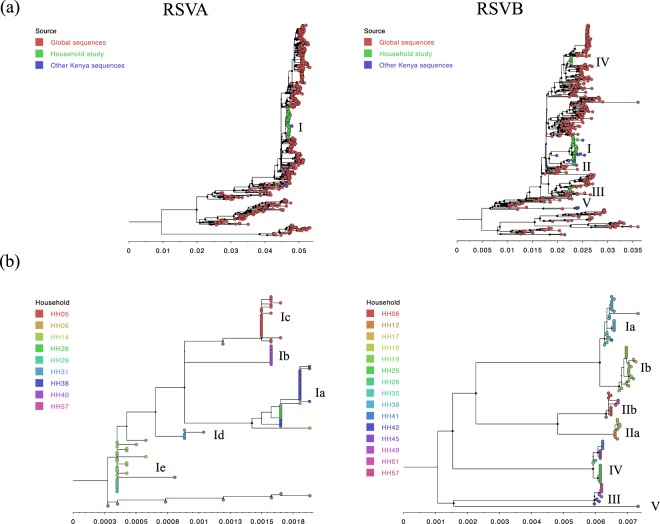
Phylogenetic relatedness of the sequenced household RSVA and RSVB genomes. (Panel a) Shows the clustering of household viruses on the global phylogeny. Taxa shapes (all provided as filled circles) of household genomes are shown in green, other available Kenyan genomes in blue and global sequences in red. The clade assignment is shown to the right-hand side of the taxa shapes. (Panel b) Shows clustering patterns of the households RSVA and B genomes on their own. The tip filled circles represent the individual genomes and are given the same colour if they belong to the same household. The clade and sub-clade assignments are shown to the right-hand side of the taxa shapes. Bootstraps support values for each branch are shown as filled circle node shapes in black. The size of the shapes is scaled by the support value that ranged from 0 to 100%.

From G gene phylogeny, all RSVA and RSVB viruses sequenced were genotypes GA2 and BA, respectively (results not shown). The genome-based maximum likelihood (ML) phylogenetic trees are shown in Fig. 1. The RSVA genomes formed a single monophyletic cluster on the global phylogeny while household RSVB genomes formed 5 distinct phylogenetic clusters interspersed with sequences from other global locations, Fig. 1, panel a. On their own, both RSVA and RSVB household genomes formed multiple phylogenetic clusters (several apparently genetically distinct and supported by >60% bootstrap values and we later assigned these into clades and sub-clades – see below). On the household genomes only ML tree, these clusters appeared to be mostly household specific with a few exceptions, Fig. 1, panel b.

**Figure 2:**
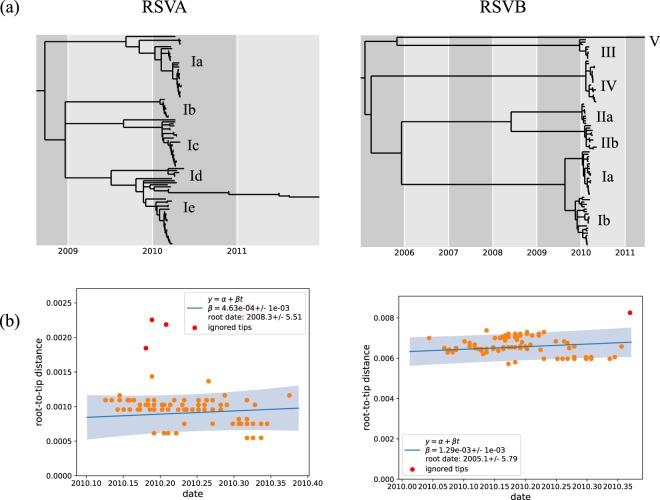
Temporal divergence characteristics of the household genomes. (Panel a) Shows a time-resolved maximum likelihood phylogenetic tree for RSVA and RSVB showing the estimated node ages and the assigned clades and sub-clades. (Panel b) Shows the strength of the phylogenetic signal in relation to sampling date for RSVA and B genomes detected in study and estimated time to the date of their most recent common ancestor (MRCA).

The time-resolved ML trees and temporal signal in nucleotide divergence of the household RSVA and RSVB viruses are shown in Fig. 2. The time to Most Recent Common Ancestor (tMRCA) estimate of all the sampled RSVB viruses was estimated to be December 2004 (Lower and Upper boundaries for 90% Highest Posterior Density (HPD) of October 1997 and September 2008), which is much earlier than the equivalent estimate for RSVA viruses of December 2008 (Lower and Upper boundaries for 90% HPD of January 1987 and December 2009), with both point estimates showing a wide uncertainty interval.

**Figure 3:**
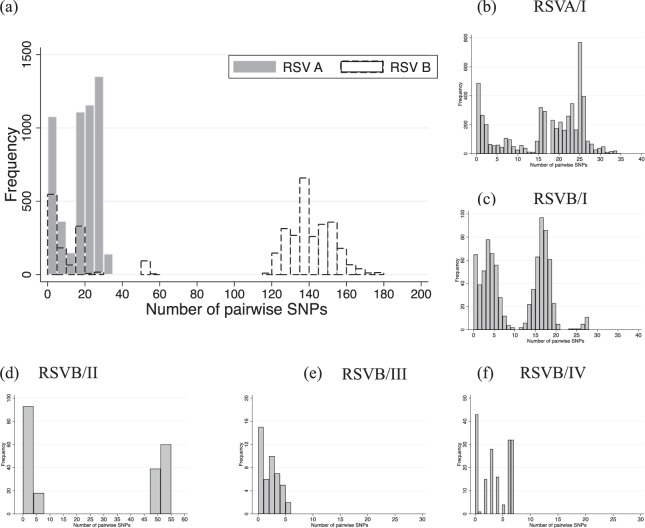
The distribution of number of pairwise SNPs of the household study viruses. (Panel a) Shows pairwise SNP count for all sequenced RSVA and B genomes. (Panel b to f) Shows the pairwise SNP count for viruses within the six clades we identified from genomic analysis of the viruses detected in the 20 households.

We quantified the genetic diversity observed within the two RSV groups by calculating the number of pairwise single nucleotide polymorphisms (SNPs) (pairwise distance) of viruses within the same group, Fig. 3, panel a. We found this value to range from 0–35 (median: 19, mean: 16.6) for RSVA and 0–177 (median: 134, mean: 99.7) for RSVB. Overall within-group pairwise distances among RSVB viruses were 6.5 times higher than those of RSVA (mean distance of 0.006094 vs 0.001065). The distribution of the number of pairwise SNPs within clusters of the household viruses observed on the global phylogeny are shown in Fig. 3, Panel b–f.

To facilitate further analysis, we assigned the household viruses into “clades” and “sub-clades” defined by both their clustering patterns on global phylogenies **(**Fig. 1, panel a), the inferred divergence dates of the strains (Fig. 2, panel a) and, the number of pairwise SNP (Fig. 3). We grouped viruses in the same clade if they occurred as a monophyletic group on the global phylogeny, had <60 pairwise SNPs across the genome with every other member of that clade and diverged more than a year prior to their date of collection. Viruses within the same clade were further assigned into sub-clades if they showed >10 pairwise SNPs differences across the genome and were estimated to have diverged more than six months prior to their date of collection (Figs 2 and 3). Using these criteria, we assigned all household RSVA strains into a single clade named RSVA/I while household RSVB strains were assigned into 5 clades named RSVB/I through RSVB/V. Viruses within clade RSVA/I were assigned into five sub-clades; RSVA/Ia through RSVA/Ie, viruses within RSVB/I clade were assigned into two sub-clades RSVB/Ia and RSV/Ib, and viruses within RSVB/II were assigned into two sub-clades RSVB/IIa and RSV/IIb.

### Virus transmission within and between households

**Figure 4:**
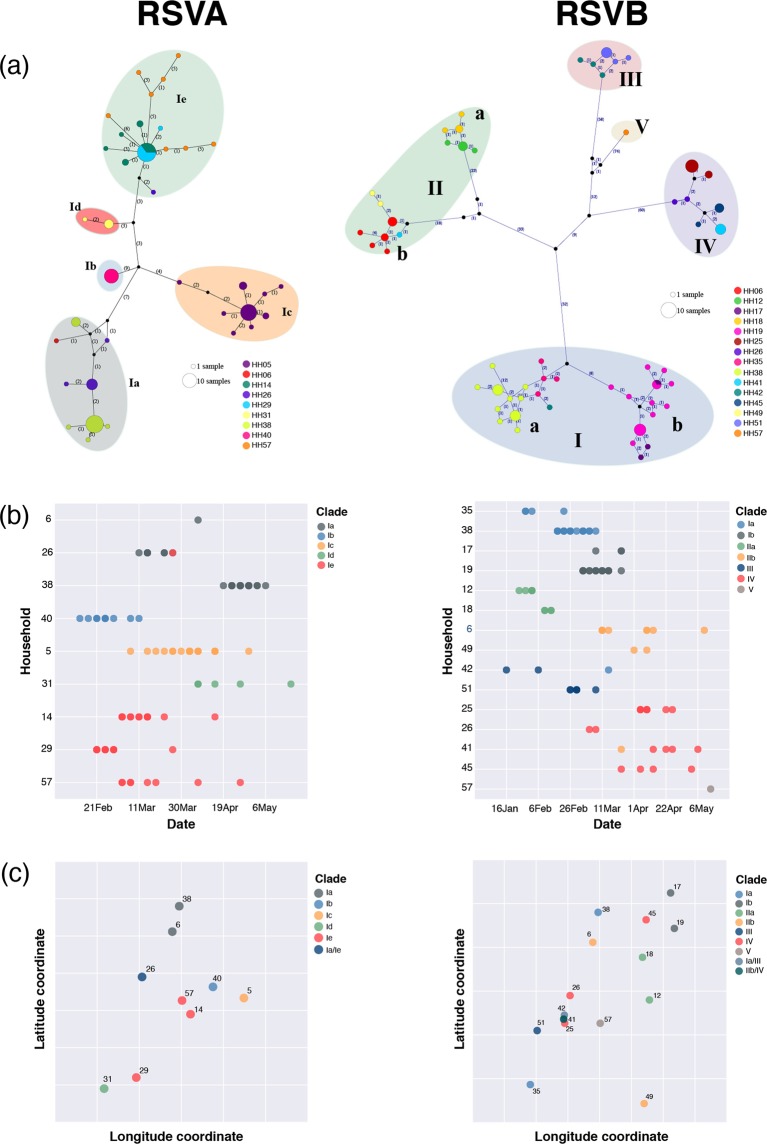
The genetic-spatio-temporal patterns of the RSVA and RSVB viruses identified from households. (Panel a) Shows the POPART minimum spanning networks of viruses within household and across households for RSVA (n = 103) RSVA and RSVB (n = 88). Each coloured vertex represents a sampled viral haplotype, with different colours indicating the different households of origin. The size of the vertex is relative to the number of sampled isolates. Numbers along each edge indicate the number of mutations. Small black circles within the network indicate unobserved internal nodes; (Panel b) illustrates the timeline of virus identified in each household, coloured by virus clade or sub-clade; (Panel c) illustrates the relative spatial locations of household with RSVA circulating with household coloured by virus clades (the longitude and latitude coordinates are removed from both axes for confidentiality). The latitude of household 42 was jittered slightly to better visualize the difference of virus clades circulating in this household and the HHs close by (HH41 and HH25).

We investigated the genomics and temporal and spatial patterns of RSVA and RSVB virus clades observed within and between households. An analysis using minimum spanning network which depicts shared differences without regard to an evolutionary model was used to detect patterns in the RSVA and RSVB genomes and examine potential intra- and inter-household transmission patterns (Fig. 4, panel a). Similar to the ML phylogenies, the majority of the viruses clustered by household with the major clusters corresponding to the clades and sub-clades observed in the ML trees. Notably clades/sub-clades RSVA/Ia, RSVA/Ie, RSVB/Ia, RSVB/Ib, RSVB/IIa, RSVB/IIb, and RSVB/IV were observed in multiple HHs indicating potential transmission linkage of the involved HHs during the epidemic. In the timeline of viruses identified (Fig. 4, panel b), all except five households (HH06, HH26, HH38, HH41 and HH42) had a single RSV clade sequenced. The exceptional households had two virus clades infecting members but mostly one of the two clades predominated e.g. in HH06, HH41 and HH42. On the other hand, in the remaining two households distinct RSVA and RSVB outbreaks occurred: HH38 in which the first outbreak was RSVB/I and at a later date a second outbreak of RSVA/I, and HH26 with concurrent RSVA/I and RSVB/IV.

The relationship between the geographical distance between the households and the RSVA and RSVB clades that circulated in these households is shown in Fig. 4, panel c. Paradoxically, some of the households that were in very close proximities experienced infections with viruses from different clades or sub-clades e.g. HH41 and HH42 were <30 meters apart, yet none of the virus clades circulating in these 2 households were shared (Fig. 4, panel c). In contrast, HH35 and HH38, separated by a distance of ~3 kilometres, shared the same virus clade (RSVB/Ia). There was no apparent correlation between inter-HH distance and genetic relatedness or between sampling dates and virus transmission, i.e. no correlation between geo-temporal-spatial patterns of virus transmission within and between households.

### Intra-host, inter-host and inter-house virus variation

**Figure 5:**
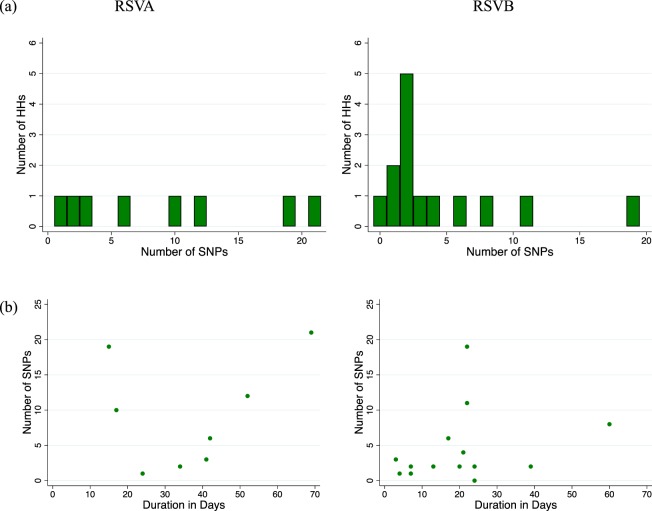
Patterns of intra-host consensus level SNPs during individual infection episodes. (Panel a) Shows the distribution of the number of SNP positions where we sequenced ≥2 genomes from the same individuals. (Panel b) Shows the relationship between the number SNPs observed and the duration between the first and last sequenced samples for these individuals. Both result panels are stratified by RSV group.

**Figure 6:**
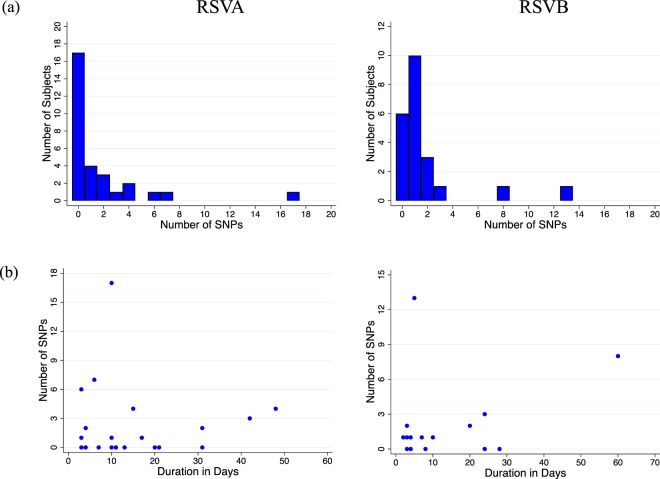
Patterns of consensus level single nucleotide polymorphism (SNP) within households during the outbreaks. (Panel a) Shows the distribution of the number of SNP position for the genome sequenced households stratified by RSV group. (Panel b) Shows the relationship between the number SNPs observed and the duration between the first and last sequenced samples for these households stratified by RSV group.

**Figure 7:**
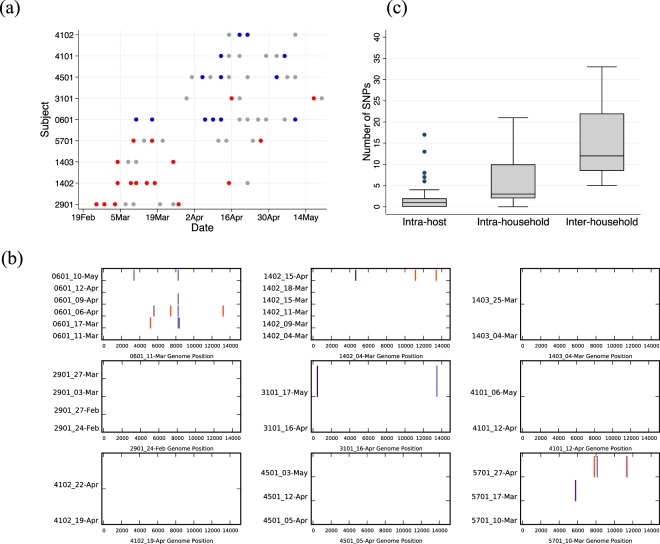
Infection and SNP patterns in prolonged virus shedders and overall SNP abundance at various levels of observations. (Panel a) Shows the patterns of positive samples (coloured grey if no sequence was obtained, red if an RSVA genome recovered and blue if an RSV B genome recovered) across time for the nine individuals who shed virus for more than 3 weeks. Note that for subject 4102 the samples available for genome comparison were only three days apart (although the individual was RSV positive for >3 weeks). (Panel b) Shows the nucleotide differences between viruses detected from these individuals. The virus sequences were compared to the earliest virus sequence from the individual. Vertical coloured bars show the nucleotide differences. Red is a change to T, green is a change to A, black is a change to G and blue is a change to C. Grey is a change to a deletion or a non-sequenced portion of the genome. (Panel c) Overall SNP abundance at various levels of observations.

**Figure 8:**
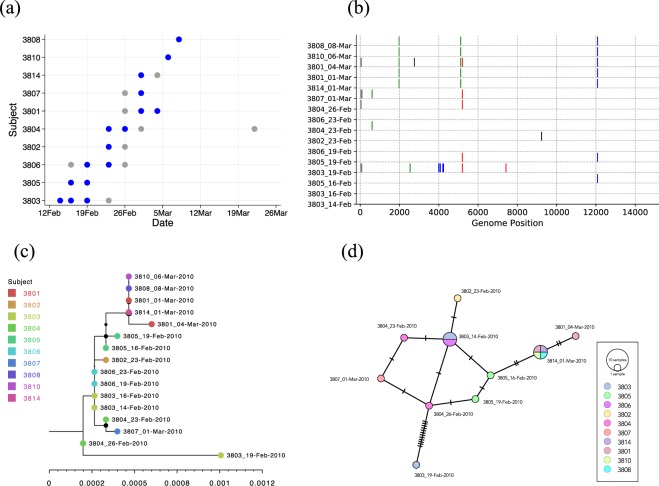
Example infection and genomic patterns of an RSV household outbreak. Here we show RSVB infection patterns in household 38 (HH38) in which 10/23 members got infected. (Panel a) Shows the patterns of positive samples across time for the 10 infected individuals. The circles represent positive samples and are coloured blue if we obtained genomes and grey if genomes were unavailable. (Panel b) Shows the nucleotide differences between viruses detected from this household. The viruses were compared to the earliest virus genome sequenced from the household. Vertical coloured bars show the nucleotide differences. Red is a change to T, green is a change to A, black is a change to G and blue is a change to C. Grey is a change to a deletion or a non-sequenced portion of the genome. (Panel c) Shows a maximum likelihood (ML) tree of all 16 genomes from the HH. The different taxon colour symbols indicate the different household members. (Panel d) Shows the POPART minimum spanning network plot of the sequences from the households. Each vertex presents a sampled viral haplotype, with different colours indicating different individuals who provided the sample. The size of each vertex is relative to the number of sampled isolates. Hatch marks indicate the number of mutations along each edge.

The SNP abundance in samples collected from same host during repeat visits and in presumed single household outbreaks are shown in Figs 5 and 6. Overall, intra-host SNPs ranged from 0–17 (median: 1, mean: 1.75, Fig. 5) while intra-household SNPs ranged from 0 to 21 (median: 3, mean: 6.2, Fig. 6). Nucleotide changes were, in general, rare intra-host during the shedding period of a presumed single episode. When changes were evident, they were usually multiple SNPs occurring simultaneously and mostly affecting the last few positive samples collected from the subject. For nine subjects who remained virus positive for more than 21 days, we compared the recovered genome sequences to determine if these represented more than one infection (Fig. 7, panel a and b). Four of these individuals showed zero change despite the sequenced samples spanning a period of over a month. For the individuals that showed SNPs, these were few (<6 SNPs). In the intra-household analysis, it appeared that the households with a higher number of SNPs (>5 i.e. falling in the upper quartile) may have experienced multiple introductions of viruses from the same clade or sub-clade e.g. in HH26 for RSVA (see Additional File: Fig. S10 sample from 2605 collected on 26-Mar-2010), HH38 for RSVB (see Fig. 8, sample from 3803 collected on 19-Feb-2010).

To track independent viruses that were either introduced from elsewhere into the study area during the epidemic or were local but diverged outside the 2009/10 season we coined the word “epidemiological strain”. Genetically, viruses referred to as same epidemiological strain had <10 SNPs across their genomes and belonged to the same clade and sub-clade (where assigned). In total, we identified 12 epidemiological strains (five within RSVA and seven within RSVB) that occurred in the study area during the six-month surveillance, eight (66.7%) of which were observed in multiple households while four were found in a single household. For the epidemiological strains that occurred in multiple households, between 5–33 (median:12, mean 15.3) SNPs were observed across their genomes. A comparison of SNP abundance intra-host, inter-host and inter-household is provided in Fig. 7, Panel c. SNP abundance appeared to increase linearly across these three levels.

### Who infected the infant(s) in the study households?

There were 22 infants from the 20 HHs. By our diagnostics, the infant in HH18 did not get RSV infected during our surveillance period. The household-by-household time-resolved infection patterns, genome alignments, phylogenies and minimum spanning sequence networks are provided in the Additional Files S3–21. We present the infection and genomic patterns of HH38 as an example in Fig. 8. Patterns of RSVA infection in HH14 and HH38 can be found in our previous publication^10^. Following examination of the patterns from all the 20 households, the summary of our deductions on who most likely infected the infant is provided in Table 2. Overall, we could infer the single most likely individual to infect the study infant for only 19% (4/21) of the infants, Table 2. For a further 19% we identified the top two individuals who most likely to have infected the infant. Note that in HH38, the infant was infected in both the RSVA and RSVB outbreaks that occurred in this household. All except one of the suspected infant infectors were aged <12 years-old.

**Table 2:** Inferring who most likely infected the infant in the household.

HH^€^ ID	Infant ID^¥^	RSV Group	Infector Identifiable?^£^	Infector ID^£^	Comment
HH05	0502	A	No	—	Infant was the 15^th^ person to be positive in HH and carried virus identical to 10 other members.
HH05	0503	A	Equivocal^ß^	0509 or 0511	Infant carried virus identical to 0509 (a toddler, asymptomatic, non-school-going member) and 0511 (a toddler, symptomatic, non-school going member).
HH05	0504	A	Yes	0518	Infant was one of the first 2 secondary cases, virus detected identical to index case 0518 (a school-aged and school-going member).
HH05	0505	A	No	—	All samples from this infant failed sequencing.
HH06	0601	B	No	—	Infant was co-index^€^ with 0603 (a school-aged, asymptomatic, non-school going member).
HH12	1201	B	No	—	Infant was the index case.
HH14	1401	A	Equivocal	1404 or 1402	Infant virus sequence had one nucleotide difference from 1404 (the index-case, a school-aged, symptomatic, school-going member) and 1402 (a toddler, symptomatic, non-school going member).
HH17	1701	B	No	—	Sequencing failed for the positive sample from the potential index case, 1705 (adult) collected 7 days earlier.
HH19	1901	B	No	—	Infant was co-index with 1903 (a pre-schooler, mostly asymptomatic, non-school going member).
HH25	2501	B	No	—	Infant was co-index with 2504 (an adult, asymptomatic except on one visit that produced a positive sample and was a non-school going member).
HH26	2601	A	No	—	Infant was the index case.
HH29	2901	A	Yes	2904	Infant was one of the three initial secondary cases, the virus sequence was identical to that from the index case 2904 (a school-aged, school-going, symptomatic member).
HH31	3101	A	Equivocal	3103 or 3105	Infant was one of the two secondary cases in the household. The virus was introduced by 3103 (a toddler, non-school going, symptomatic) member. The other potential infector (3105) was a school-aged, school-going member, asymptomatic in 2/3 positive visits.
HH35	3501	B	No	—	Infant was co-index with 3503 (a pre-schooler member, asymptomatic in 2/3 positive visits, school going).
HH38	3801	A	Yes	3821	Infant among the first 2 secondary cases. Infant virus sequence had one nucleotide difference with the index case 3821 (an adult, asymptomatic, non-school going member).
HH38	3801	B	Yes	3805	Infant was 6^th^ positive case in HH and carried virus highly similar to the 3805 virus (a school-aged, asymptomatic, non-school going member).
HH40	4001	A	Equivocal	4004 or 4001	The virus sequence from infant was identical to the index case virus (4004, a school-aged, symptomatic and school-going member) and the first secondary case (4002, a toddler old, symptomatic and non-school going member).
HH41	4101	B	No	—	Infant was the index case in this household.
HH42	4201	B	No	—	Infant was the index case in this household.
HH45	4501	B	No	—	Infant was the third secondary case in the household, two of which yielded no sequence and the other had two nucleotide changes.
HH49	4901	B	No	—	Infant was the index case.
HH51	5101	B	No	—	Infant was the 5^th^ positive case in the household and the 5101 virus sequence was equidistant genetically from all other members.
HH57	5701	A	Equivocal	5707 or 5702	This household showed high genetic variation in general. Infant was the third secondary case in the household and virus was close to index case 5707 (a school-aged member) and 5702 (a toddler member).

^¥^Infant refers to the persons that were <1 year-old during our RSV surveillance period; Definition of the age defined intervals are: toddler (ages 1 to <3 years); pre-schooler (ages 3 to <6 years); school-aged child (ages 6 to <12 years); adolescent (ages 12 to <18years); adults (aged >18 years); ^£^Infector refers to the person whom most likely infected the infant in the household**;** ^€^Co-index cases refer to the two individual whom were found concurrently first to be RSV positive in a household. ^ß^Equivocal refers to when two individuals had an equal probability of being the source of the infant infection by our analysis approach and that could not be resolved further. Note that by our diagnostics, the infant in HH18 did not get RSV infected during our surveillance period.

## Discussion

The origin of this work was a study of who introduces RSV into the household and who infects the infant^20^. This was motivated by unsuccessful vaccines for early infants and that evaluation of other options (family cocooning, school age vaccination^20,42^) requires an improved understanding of WAIFW. Our earlier work, based on temporal case observations, clearly suggested that the older children (siblings or cousins aged <15 years), particularly those attending school, played an important role in introducing the virus into the household leading to infant infection, but was not able to resolve within household transmission chains^20^. We have subsequently formalised the epidemiological analysis of RSV transmission in the household using an individual-based statistical approach to quantify the risk of infection from a range of host, pathogen and environmental factors^7^. The present study takes an alternative perspective of the problem, by focusing on the temporal patterns of genomic sequence variation to elucidate who infects whom in the household. This work extends a smaller study based on genomes of RSVA from 9 households^10^, to the current study of genomes of RSVA and RSVB in 20 households.

Our key observation from the present analysis is that RSV consensus genomes incur zero to just a few nucleotide substitutions within infected individuals (median: one SNP per episode) or between infected individuals of the same household (median: three SNPs). Combined with the rapid spread of RSV within households and incomplete sequencing (~50%) of the positive samples challenges the reconstruction of the transmission using genomic data alone. For six households (32%) where the infant was infected (n = 8), we could identify the 1–2 most likely individuals who infected them. The infant suspected infectors were mostly household co-occupants <12 year of age (7/8, specifically toddlers (43%) and school-aged (50%) age-groups). Only in a single instance was an adult co-occupant (mother) suspected to be the infant infector. In the remaining households (13/19, 68%), the infant was identified as either the household index case, a co-index case or the sequencing of key samples failed, making it difficult to infer their infection source.

Elsewhere we attempted to utilise shared minor variants identified from deep sequencing data for RSV in these same households to draw out patterns of transmission^43^. The conclusion of the work was that shared minor variants provide little additional resolving power to discern chains of transmission beyond that possible through consensus sequences.

Previously, only two other studies focused on transmission of RSV infections within households^8,44^. In these studies, notably, it was assumed that a single infection source was responsible for the cases occurring in the same household, whereas temporally it can be difficult to fully establish this. Furthermore, without virus genotyping and, ideally, full-genome sequence data, the composition of outbreaks cannot be definitively established; as we have seen multiple concurrent virus introductions into households are not uncommon. Furthermore, for study of Heikkenen *et al*.^44^, the investigators followed up the household only after the index infant had been admitted to hospital, which limits the possibility of observing preceding transmission events including who infected them.

Our study involved sampling irrespective of symptom status, coupled to sensitive molecular diagnostics and genomic sequencing, which has given a clear indication that households are indeed a common space for RSV transmission^7^. Similar to previous studies based solely on epidemiological (not sequence) data^8,44^, we highlight the importance of the infant’s elder siblings especially those under 12 years of age as a source of the infant infection. Adults in the households played only a minor role when considered either as household RSV infection introducers or as infant infectors. Furthermore, by analysis of RSVA viruses from nine households, we had previously shown that most (6/9, 67%) RSV infections in a household outbreak result from a single introduction of the virus^10^. Here we have extended the analysis to RSVB, confirming a closely similar pattern to RSVA.

The unique household study design here allowed us to compare the phylodynamics of RSVA and RSVB viruses. Overall, the sequenced RSVB viruses showed ~7 times greater genomic diversity compared to RSVA. It is likely that the observed difference reflects annual stochasticity in the number of introduced strains rather than an inherent biological difference although a few previous reports indicated existence of subtle differences between the two groups in transmissibility and local persistence^21,45^. Despite the close genetic relatedness of RSVA viruses detected in the study, our analysis showed that the 9 infected households were invaded by up to 5 distinct RSVA “epidemiological strains” that diverged at least 6 months before their collection date. For RSVB we determined that the 14 infected households were invaded by up to 7 distinct RSVB epidemiological strains. Highly similar intra-household and intra-host genomic variation patterns were observed between the two groups.

Due to the intense logistics involved in undertaking such a study, only 50 households from one administrative unit (14,998 persons in 1,835 homesteads) within Kilifi County were recruited^20^. The genome sequencing work targeted 20 households where ≥2 members were found to be RSV infected. Despite these households occurring in a small geographical area (~20 km^2^) it was surprising to see up to 12 epidemiological strains in circulation. Most of the sampled viruses clustered by household. Some households shared the infecting strain with other households, suggesting a shared infection source although direct transmission between these households was unlikely given the large fraction of non-sampled households. Four out of the 12 identified epidemiological strains occurred only in one household each. Notably, households in close physical proximity did not necessarily end up being infected with similar virus clades or subclades implying other unobserved epidemiological factors rather than physical proximity may be more important in determining WAIFW in this community^46^.

Our earlier epidemiological analysis suggested school-going house-members are the sub-population (39%) most likely to introduce the infection into the household^20^. Perplexingly, the study infants were the second most frequent index cases (32%) and were co-index in a further 14% of the household episodes^20^. It is possible that some of the infant co-index cases were the infectors of infants in the household, but our diagnostic method (nasopharyngeal swab combined with RT-PCR) failed to detect the virus in the preceding samples. This may occur perhaps due to limited virus replication in older individuals or our 3–4 days sampling interval may have been too wide to capture index cases before onward transmission. By our diagnostic method, a parent was the index case only in one household.

It was surprising to find few to no SNPs in RSV genomes from individuals appearing to shed RSV for up to 2 months. These individuals may have been true prolonged shedders of the virus or were virus re-infected. If prolonged shedders, then it is perplexing that in some individuals, there was one or more negative sample(s) separating the positive samples. Alternately, these could be false negative assay results which may have arisen due to the sensitivity of our sampling or diagnostic method or that the virus was temporarily absent from the upper respiratory tract airway but was still present elsewhere in the individual’s respiratory tract. Prolonged shedding of RSV of up to 2 months has been previously reported especially in immune-compromised populations^47,48^. Alternatively, if these were indeed reinfections, then this observation calls for an interrogation of protective RSV immune responses and this has implications to the development of effective RSV vaccines^49,50^.

Our study illustrates both the value and the limitations of RSV genomic data in tracking transmission of this rapidly spreading infection in a household setting. The pace of RSV substitutions was demonstrated to be insufficiently fast to enable the full inference of within household RSV transmission trees. Additionally, we have previously shown that patterns of sharing of minor variants does not add insight beyond the consensus sequence approach^43^. Since in close to half of the study households the infant participant was the infection index or co-index case, for future studies we recommend sampling protocols that also consider, in addition to households, other potential RSV transmission settings in the community e.g. child-care centres, post-natal clinics, schools, school transportation, sporting events etc. Contact data should be collected to reinforce the viral sequence data and epidemiological data to support robust inferences of transmission pairs^46^. The protocols for genomic sequencing also need to be optimised to obtain virus sequences even from samples with diminishing virus titres. Given the imperfections of analyses of epidemiological data or genomic data in isolation, there is a clear need to undertake the joint analysis of both sources of information using a probabilistic framework^7^, that will allow inference of events not directly observable with inevitably imperfect data.

## Supplementary information

Additional File

Dataset 1

## Data Availability

The sequence data from this study has been deposited in both GenBank and Short Read Archive databases (see accession details in Supplementary Dataset). For more detailed information beyond the metadata used in the paper, there is a process of managed access requiring submission of a request form for consideration by our Data Governance Committee (http://kemri-wellcome.org/about-us/#ChildVerticalTab_15).
