# Supplementary material for: Genomic analysis of respiratory syncytial virus infections in households and utility in inferring who infects the infant
Source: Sci Rep. 2019 Jul 11;9:10076. doi: 10.1038/s41598-019-46509-w (PMC6624209; doi:10.1038/s41598-019-46509-w)
Supplement: Supplementary file 1 — Additional File [file 41598_2019_46509_MOESM1_ESM.pdf]

# ADDITIONAL FILE

## Genomic analysis of respiratory syncytial virus infections in households and utility in inferring who infects the infant

Charles N. Agoti[1, 2, *], My V. T. Phan [3, β], Patrick K. Munywoki[1, $], George Githinji[1], Graham F. Medley[4], Patricia A. Cane[5], Paul Kellam[3, 6], Matthew Cotten[3, #] and D James Nokes[1, 2, 7]

*[1]Kenya Medical Research Institute (KEMRI) – Wellcome Trust Research Programme, Epidemiology and Demography Department, Kilifi, Kenya. [2]Pwani University, School of Health and Human Sciences, Kilifi, Kenya. [3]Wellcome Trust Sanger Institute, Cambridge, United Kingdom. [4]London School of Hygiene and Tropical Medicine (LSHTM, Department of Global Health and Development and Centre for Mathematical Modeling of Infectious Disease, London, United Kingdom. [5]Public Health England, Porton Down, Salisbury, United Kingdom. [6]Imperial College London, Department of Infection, London, United Kingdom .[7]University of Warwick, School of Life Sciences and Zeeman Institute, Coventry, United Kingdom.*
*[β]Current address: Erasmus Medical Center, Department of Viroscience, Rotterdam, The Netherlands. [$]Current address: Center for Disease Control and Prevention, Division of Global Health Protection, Nairobi, Kenya. [#]Current address: MRC/UVRI & LSHTM Uganda Research Unit, Entebbe, Uganda, and MRC-University of Glasgow Centre for Virus Research, Glasgow, UK.*

[*]Corresponding author (email: cnyaigoti@kemri-wellcome.org)

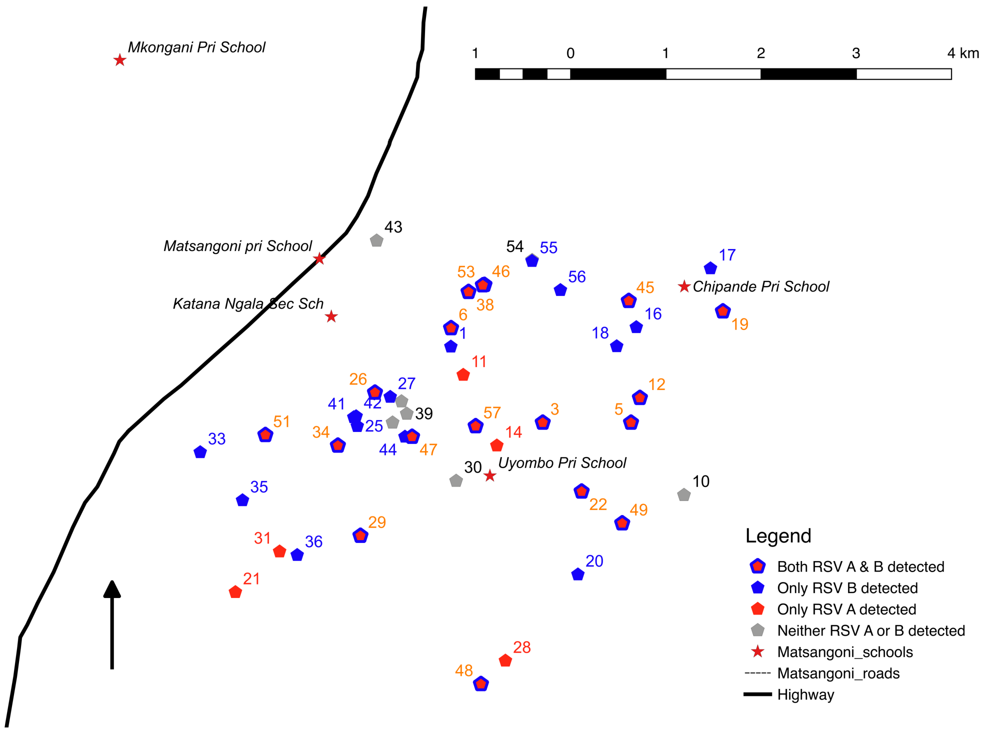


**S1 Figure.** The spatial distribution of the study households and the RSVA and B RT-PCR detection status for each of them.

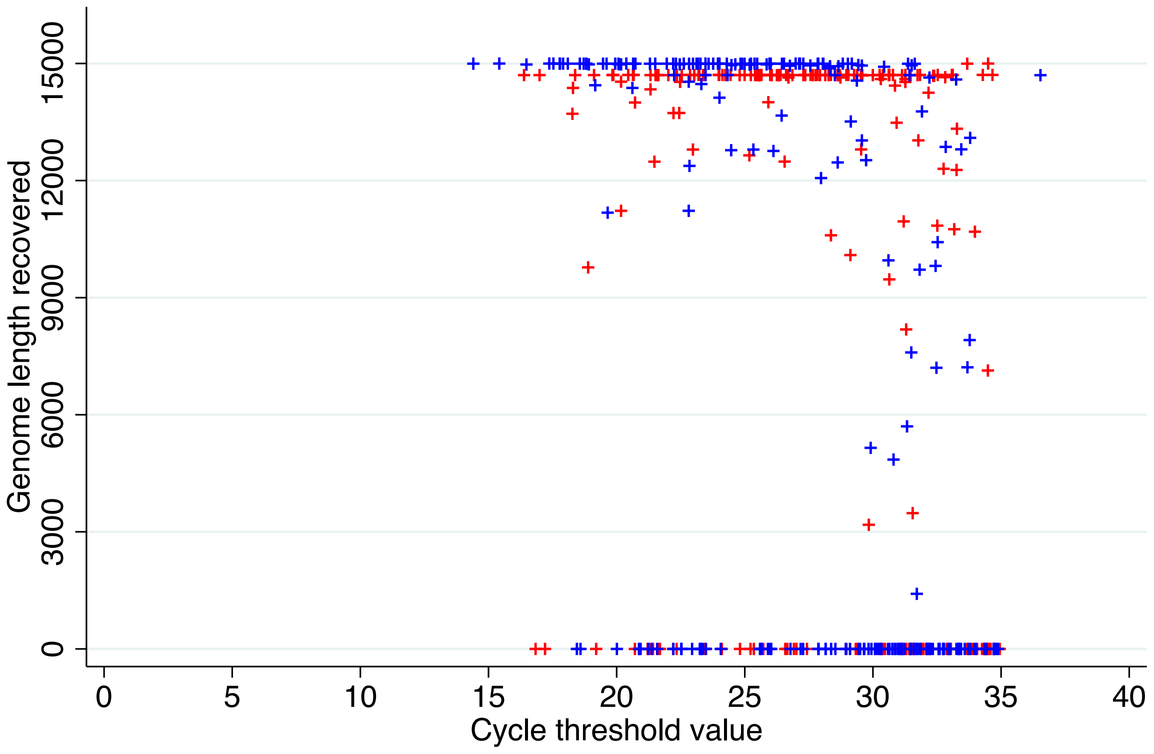


**S2 Figure.** The relationship of recovered genome length and the diagnostic real-time RT-PCR cycle threshold (Ct), a marker of the virus titre in the sample. RSVA  shown by red markers while RSVB is shown by blue markers.

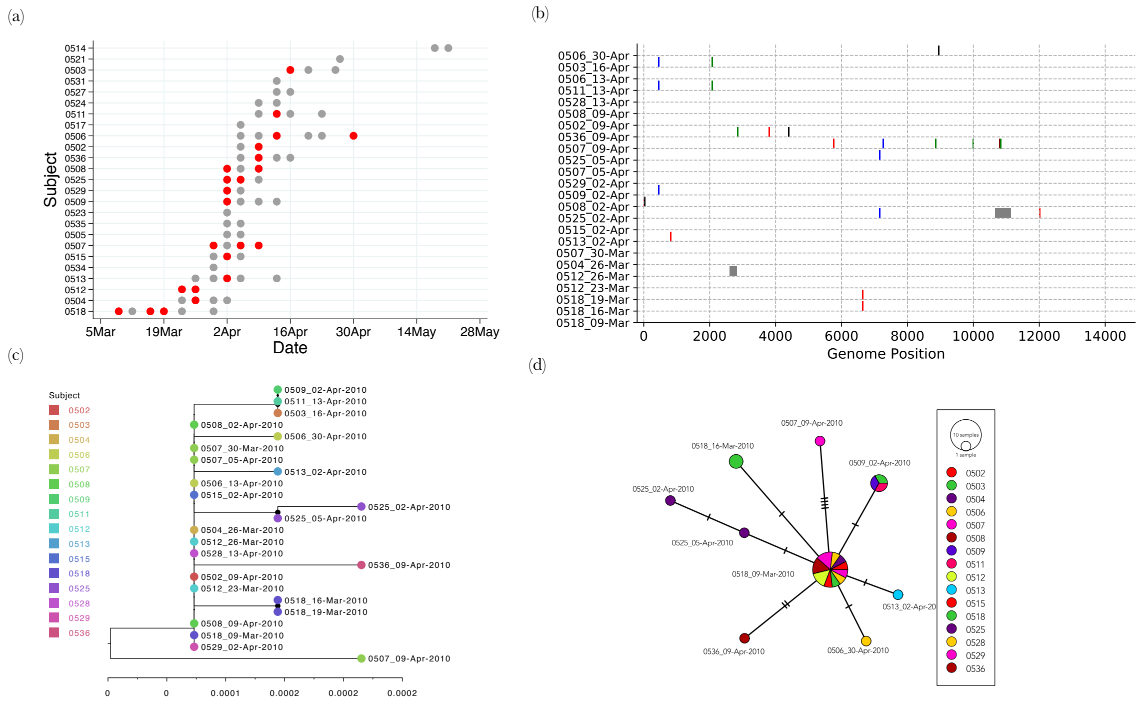


**S3 Figure.** Infection and genomic patterns of RSVA outbreak in household five (HH05) in which 25/37 members got infected. *Panel A* shows the patterns of positive samples across time for the 25 infected individuals. The circles represent positive samples and are colored red if we obtained genomes and grey if genomes were unavailable. *Panel B* shows the nucleotide differences between viruses detected from this household. The viruses were compared to the earliest virus genome sequenced from the household. Vertical coloured bars show the nucleotide differences. Red is a change to T, green is a change to A, black is a change to G and blue is a change to C. Grey is a change to a deletion or a non-sequenced portion of the genome. *Panel C* shows a maximum likelihood (ML) tree of all 24 genomes from the HH. The different taxon colour symbols indicate the different household members. *Panel D* shows the POPART minimum spanning network plot of the sequences from the households. Each vertex presents a sampled viral haplotype, with different colours indicating different individuals who provided the sample. The size of each vertex is relative to the number of sampled isolates. Hatch marks indicate the number of mutations along each edge. An infant shape is indicated next to the subject who was < 1 year old.

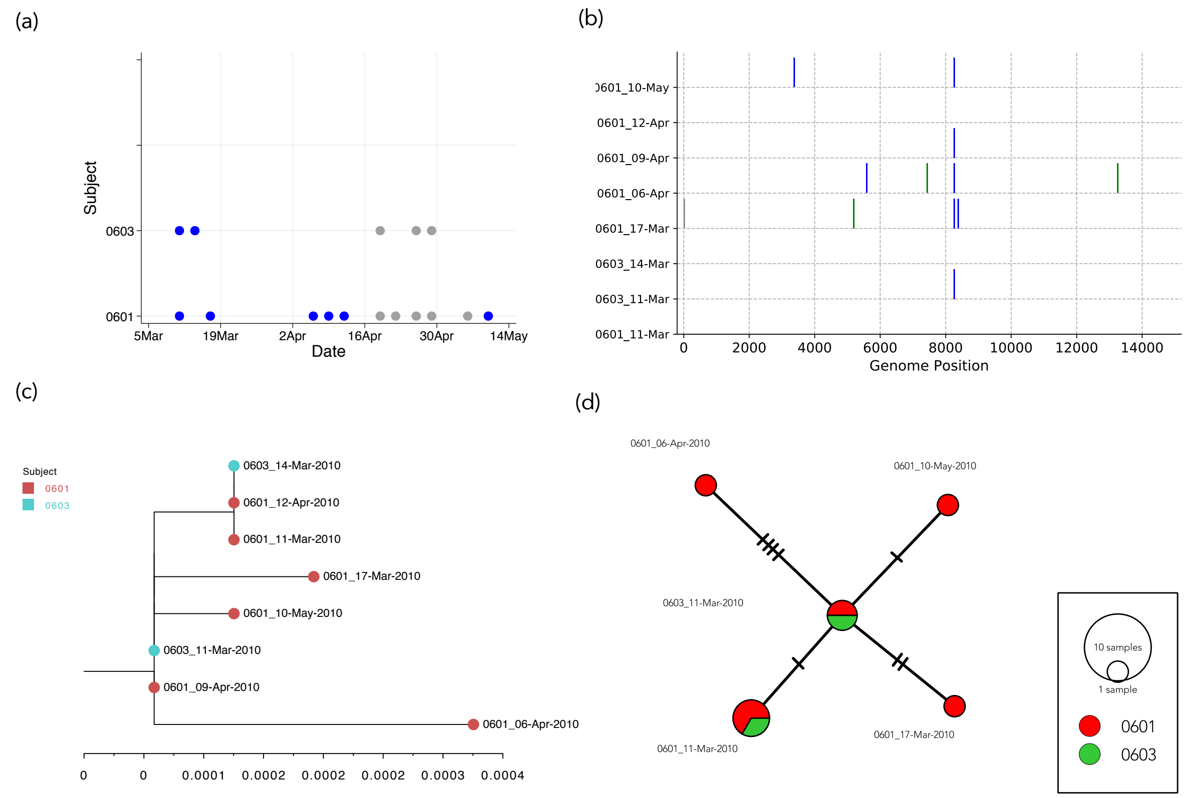


**S4 Figure.** Patterns of RSVB infection in household six (HH06) in which 2/6 members got infected. The legend details for *Panel A-D* are same as S3 Figure.

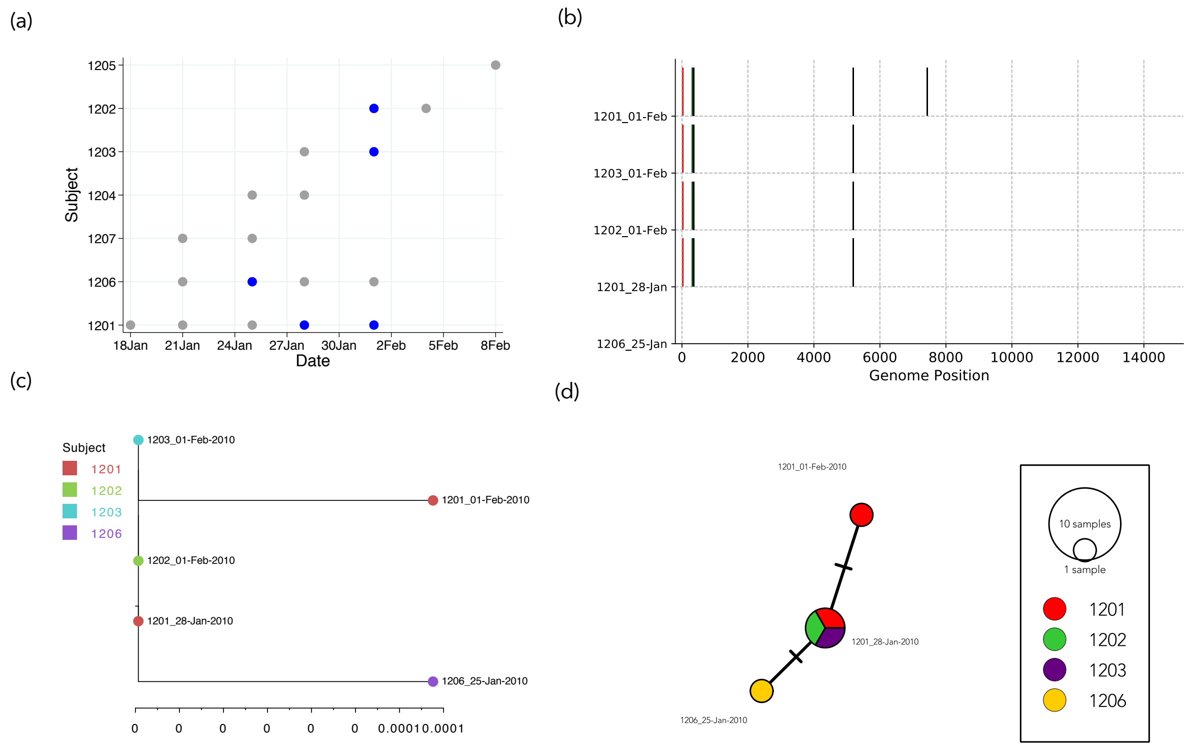


**S5 Figure.** Patterns of RSVB infection in household 12 (HH12) in which 7/20 members got infected. Sequencing failed for all samples from 3/7 infected members. The legend details for *Panel A-D* are same as S3 Figure.

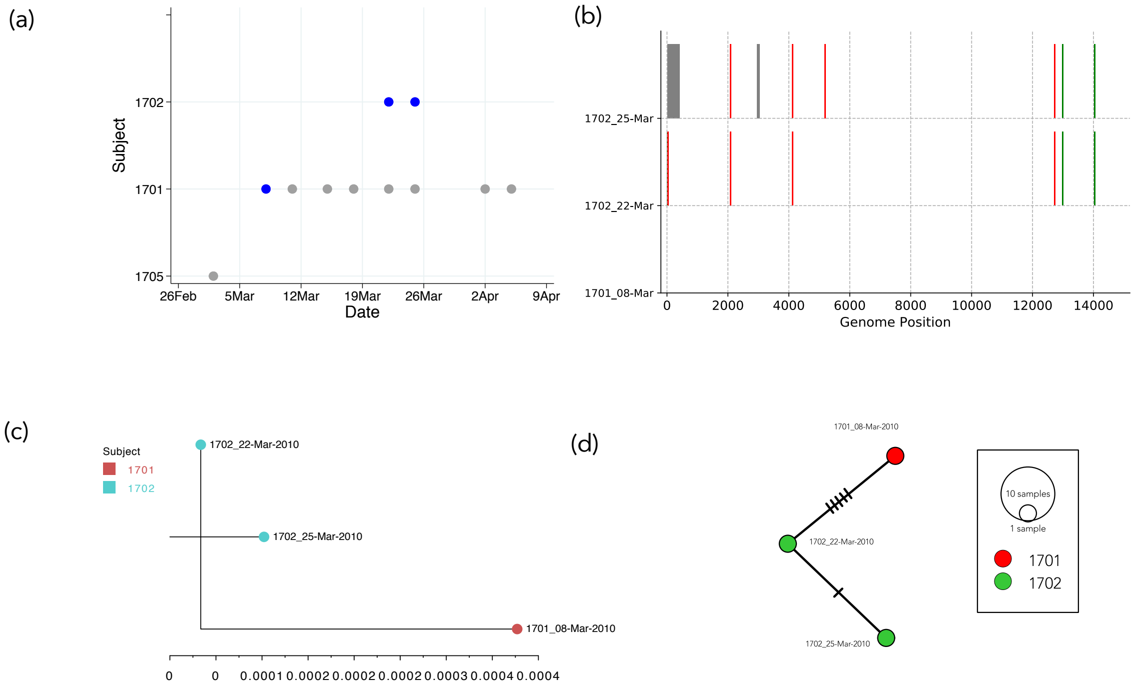


**S6 Figure.** Patterns of RSVB infection in household 17 (HH17) in which 3/5 members got infected. One of the infected members had a single positive sample that also failed sequencing. The legend details for *Panel A-D* are same as S3 Figure.

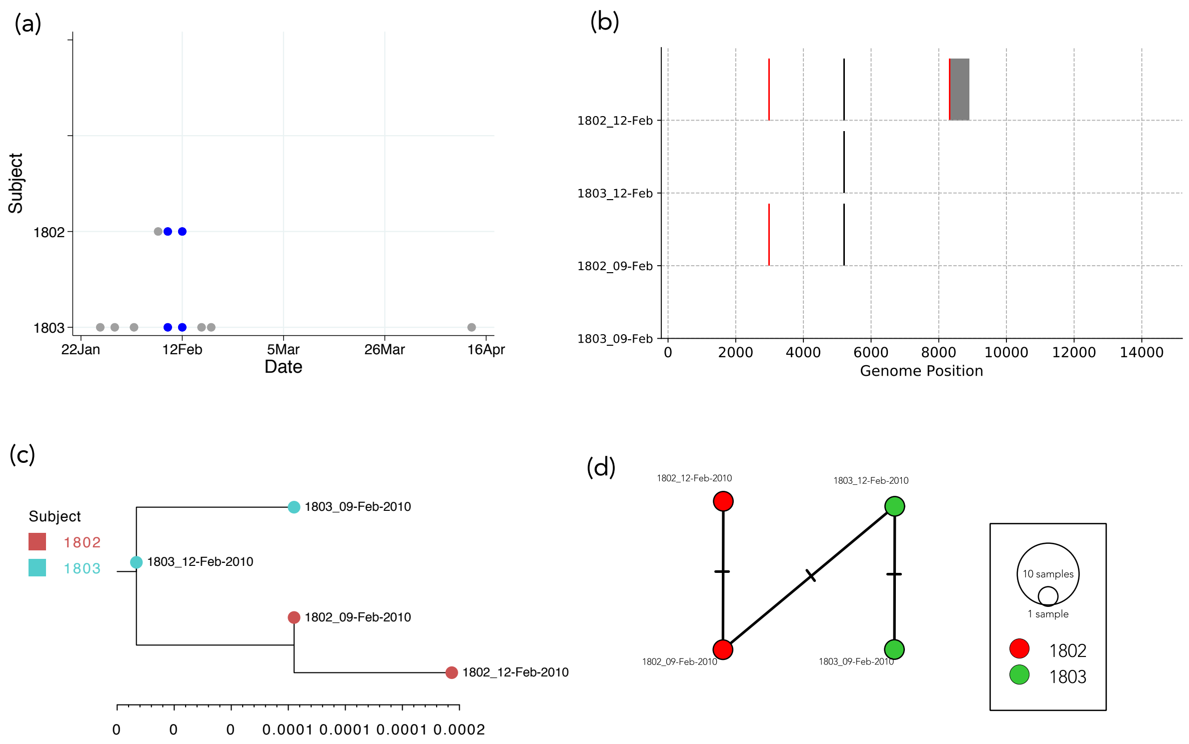


**S7 Figure.** Patterns of RSVB infection in household 18 (HH18) in which 2/8 members got infected. The legend details for *Panel A-D* are same as S3 Figure.

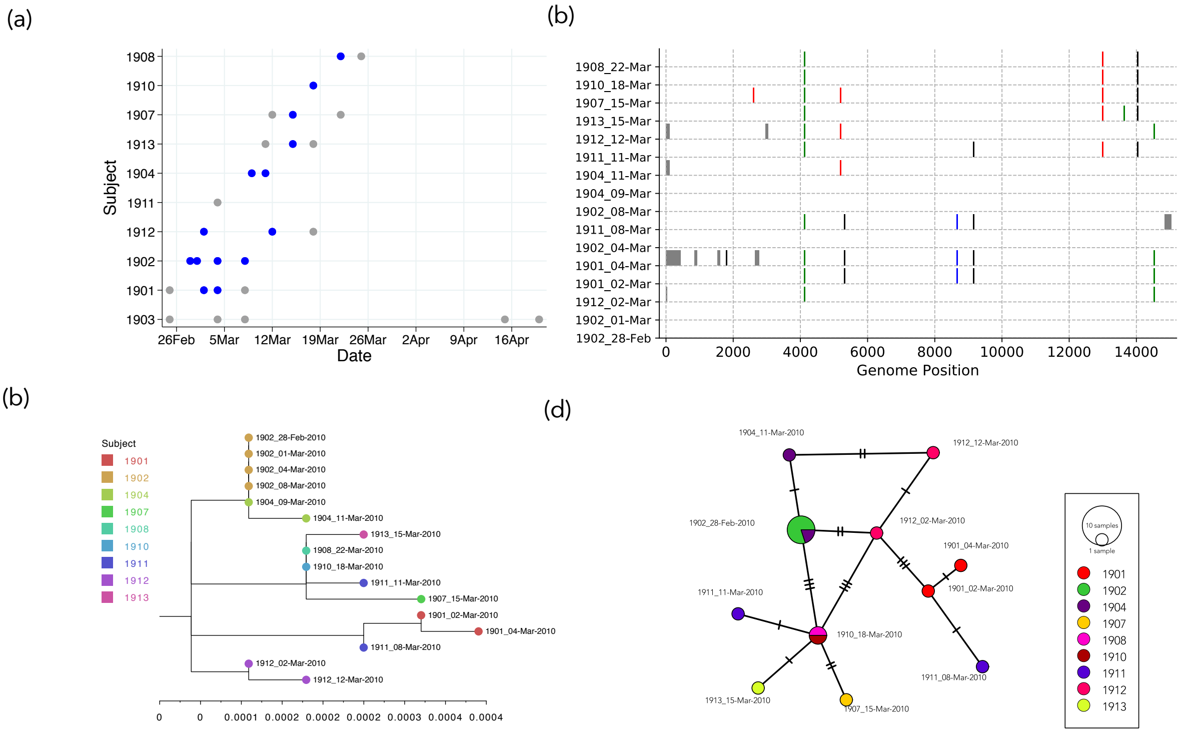


**S8 Figure.** Patterns of RSVB infection in household 19 (HH19) in which 10/14 members got infected. Two of the infected members failed sequencing in all samples collected from them. The legend details for *Panel A-D* are same as S3 Figure.

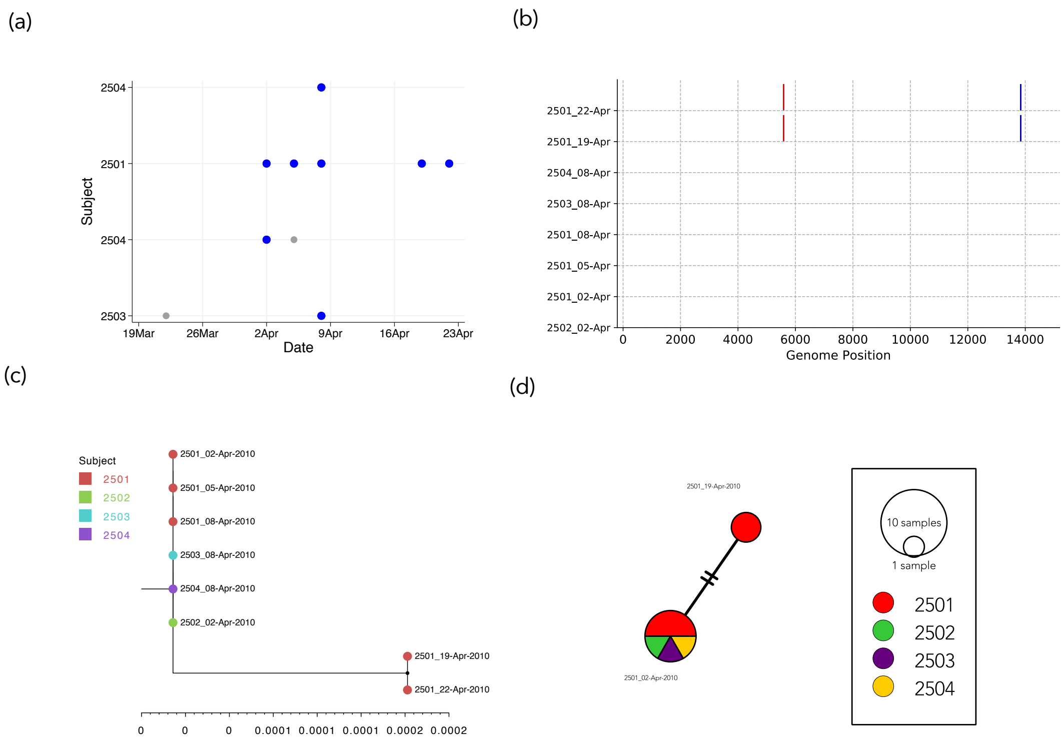


**S9 Figure.** Patterns of RSVB infection in household 25 (HH25) in which all (4/4) members got infected. The legend details for *Panel A-D* are same as S3 Figure.

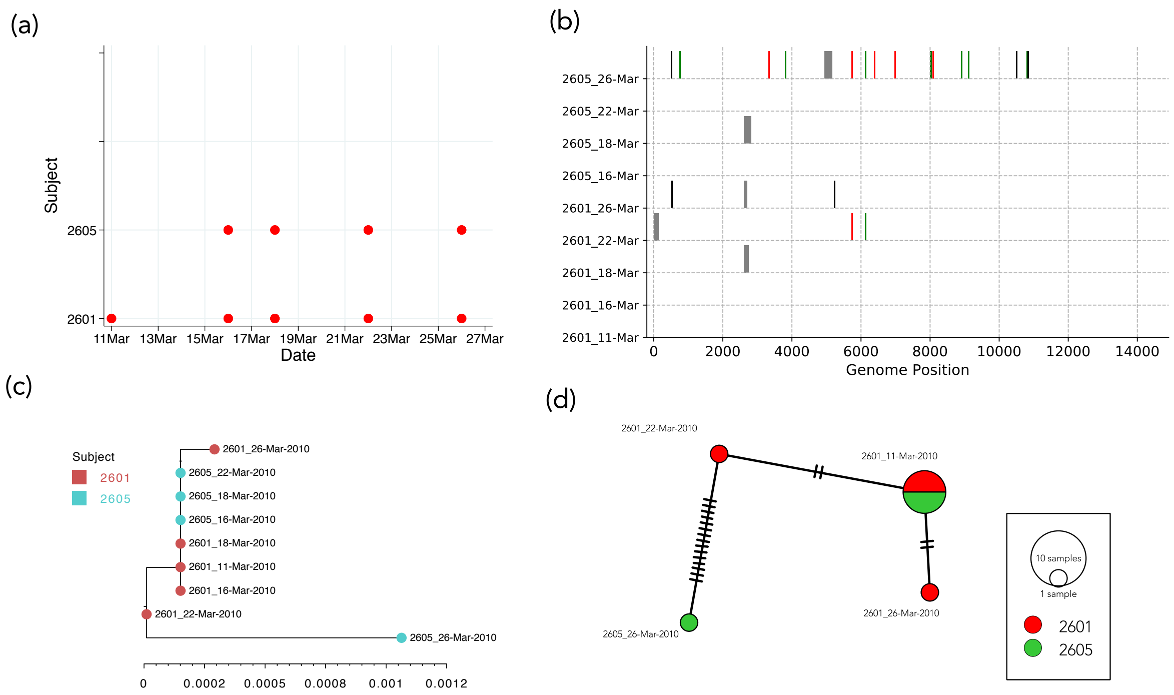


**S10 Figure.** Patterns of RSVA infection in household 26 (HH26) in which 2/5 members got infected. All the RSVA samples from this household successfully sequenced. The legend details for *Panel A-D* are same as S3 Figure.

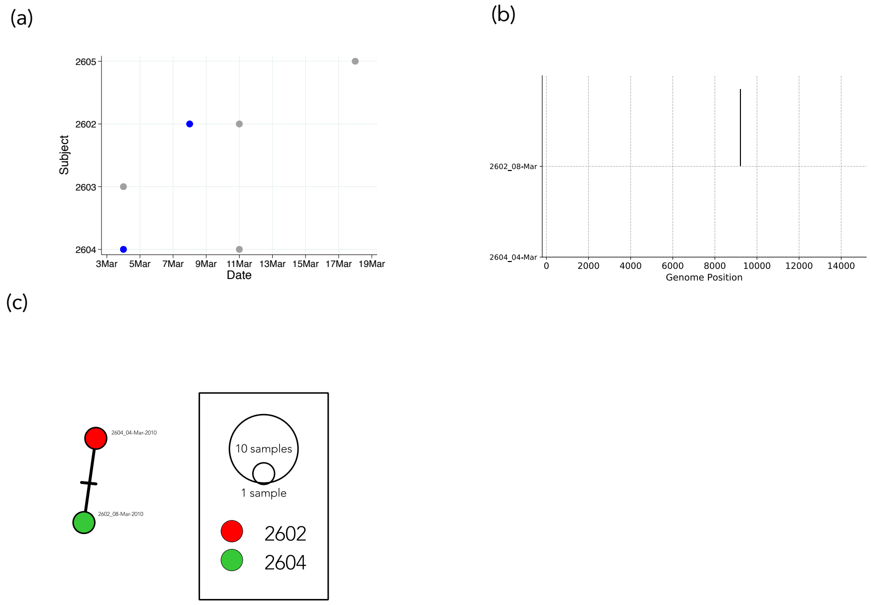


**S11 Figure.** Patterns of RSVB infection in household 26 (HH26) in which 4/5 members got infected. Two of the infected members failed sequencing in all samples collected from them. The legend details for *Panel A-D* are same as S3 Figure.

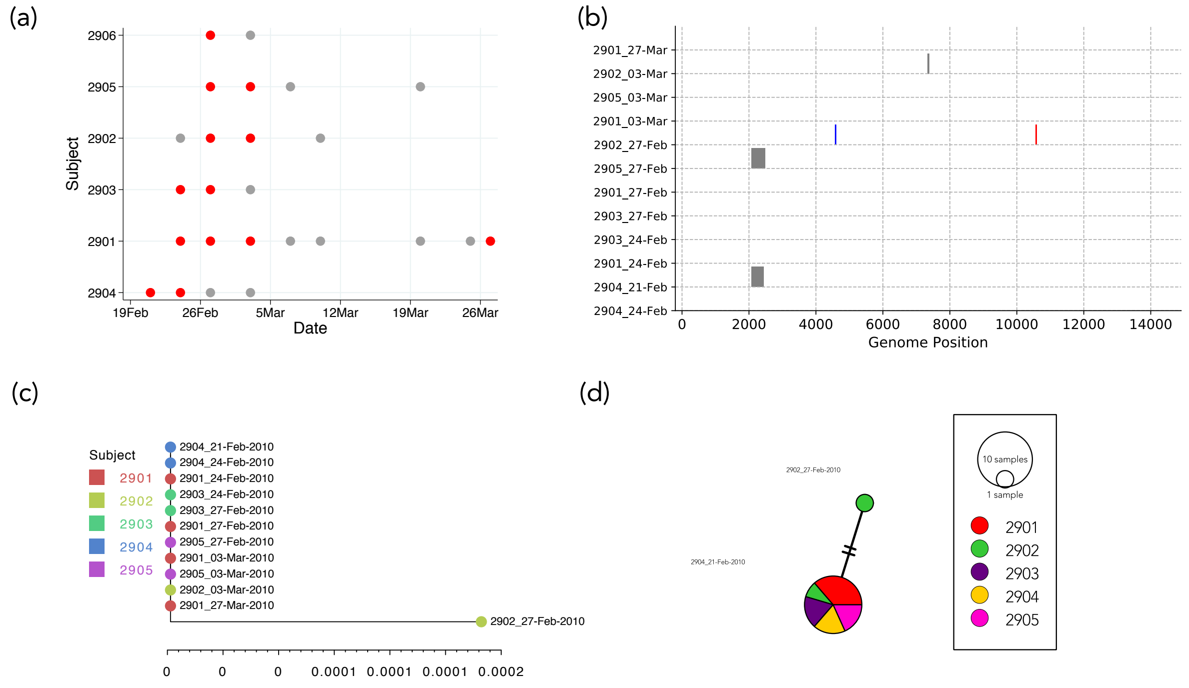


**S12 Figure.** Patterns of RSVA infection in household 29 (HH29) in which 6/7 members got infected. The legend details for *Panel A-D* are same as S3 Figure.

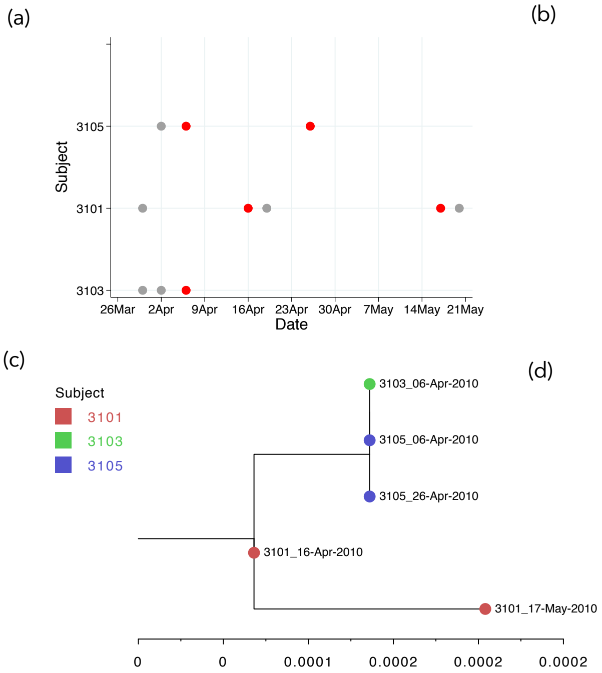


**S13 Figure.** Patterns of RSVA infection in household 31 (HH31) in which 3/11 members got infected. The legend details for *Panel A-D* are same as S3 Figure.

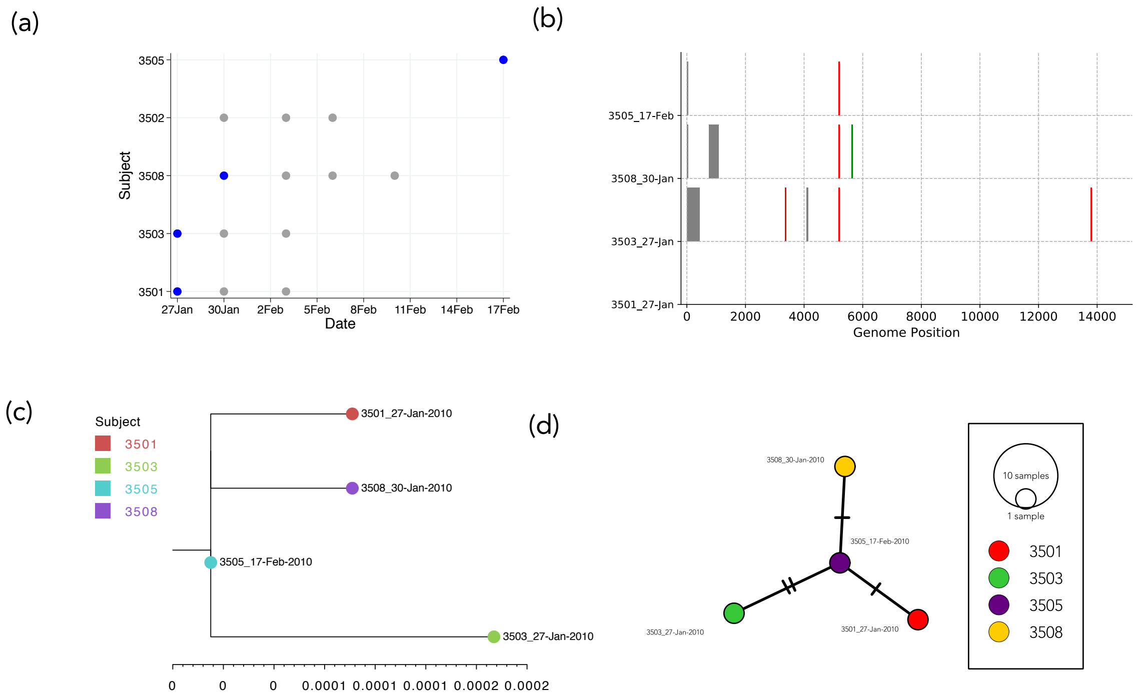


**S14 Figure.** Patterns of RSVB infection in household 35 (HH35) in which 5/8 members got infected. One of the infected members failed sequencing in all positive samples collected from them. The legend details for *Panel A-D* are same as S3 Figure.

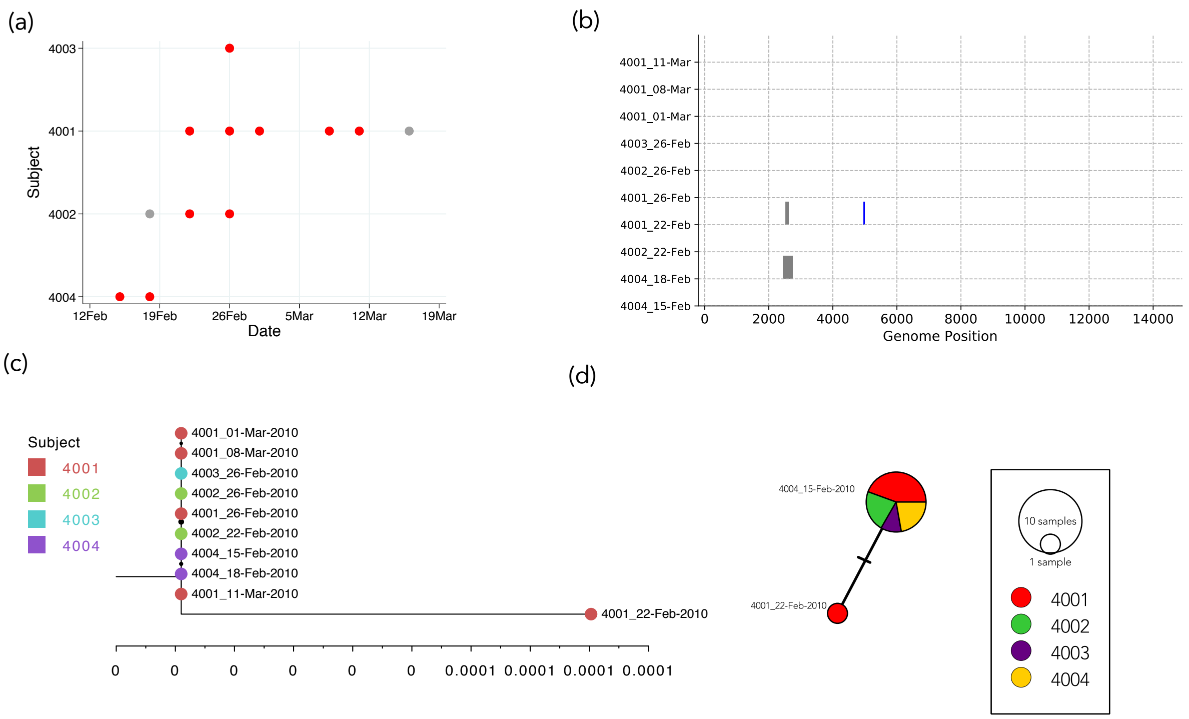


**S15 Figure.** Patterns of RSVA infection in household 40 (HH40) in which 4/5 members got infected. The legend details for *Panel A-D* are same as S3 Figure.

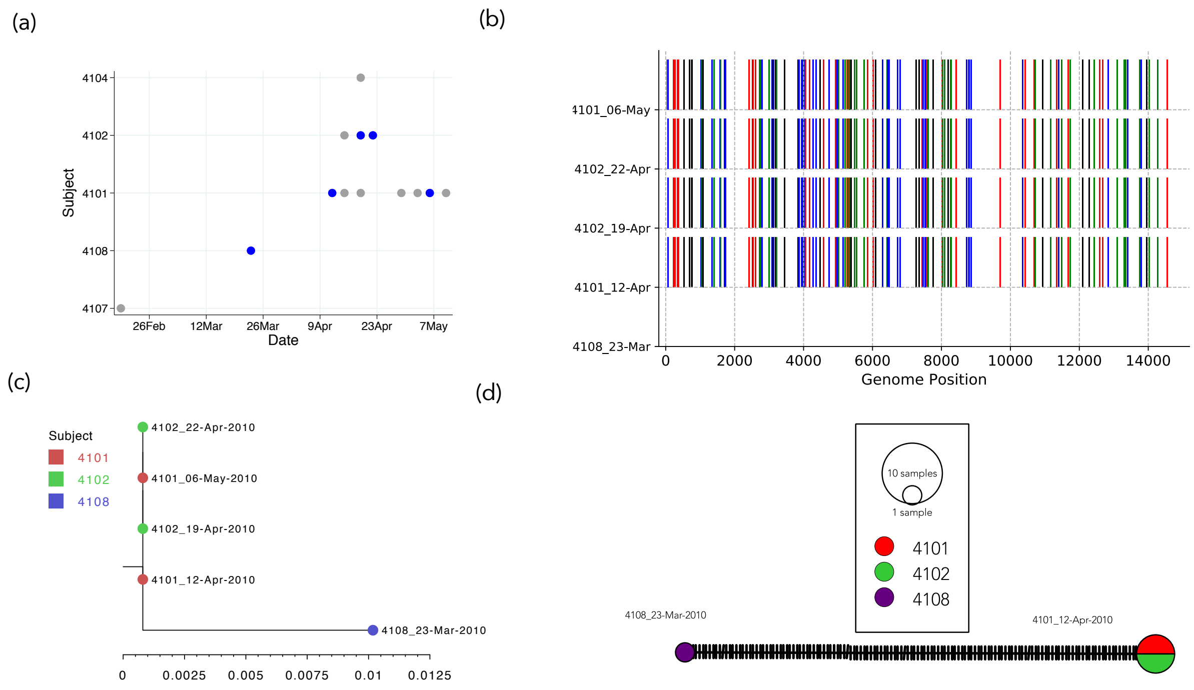


**S16 Figure.** Patterns of RSVB infection in household 41 (HH41) in which 4/8 members got infected. One of the infected members had a single positive sample that also failed sequencing. The legend details for *Panel A-D* are same as S3 Figure.

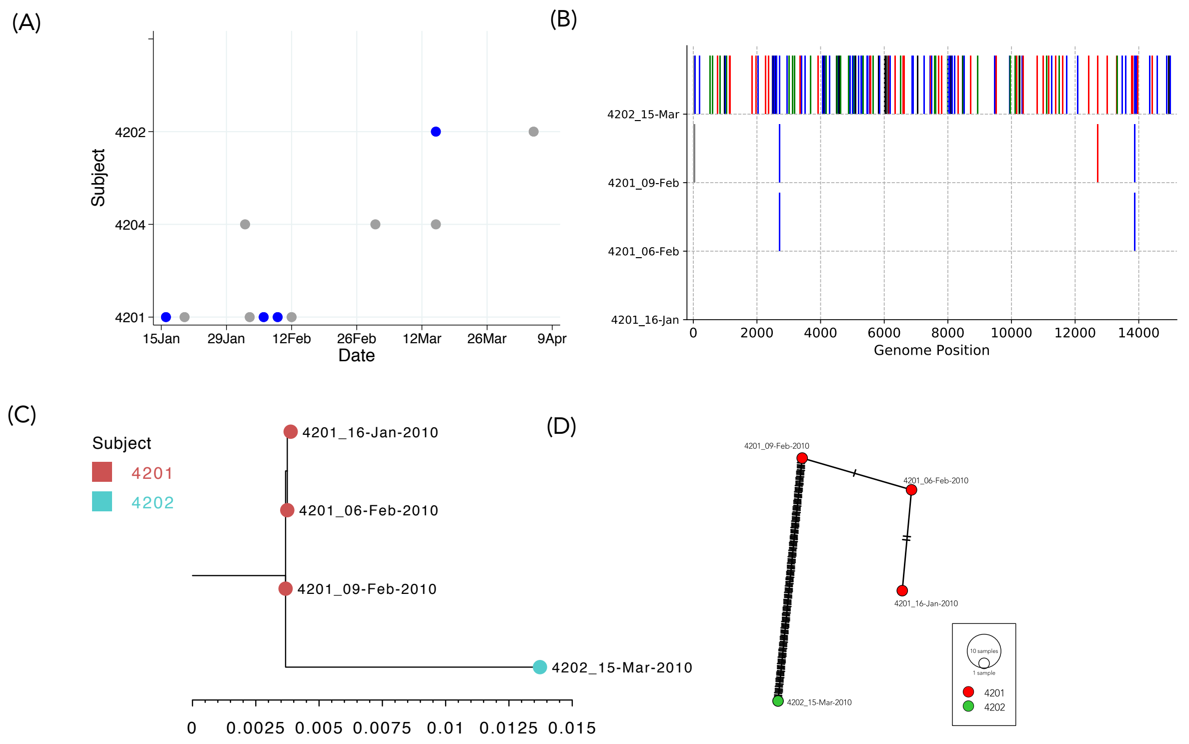


**S17 Figure.** Patterns of RSVB infection in household 42 (HH42) in which 3/6 members got infected. For one of the infected members all three positive samples failed sequencing. The legend details for *Panel A-D* are same as S3 Figure.

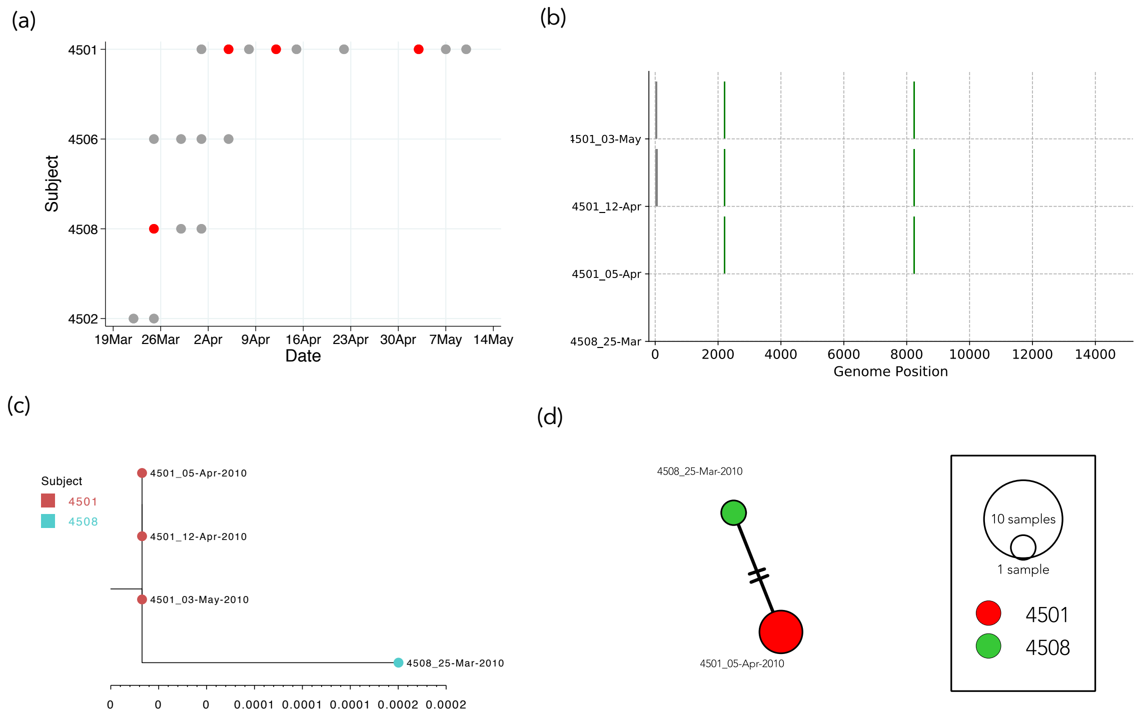


**S18 Figure.** Patterns of RSVA infection in household 45 (HH45) in which 4/10 members got infected. For two of the infected members all their positive samples failed sequencing. The legend details for *Panel A-D* are same as S3 Figure.

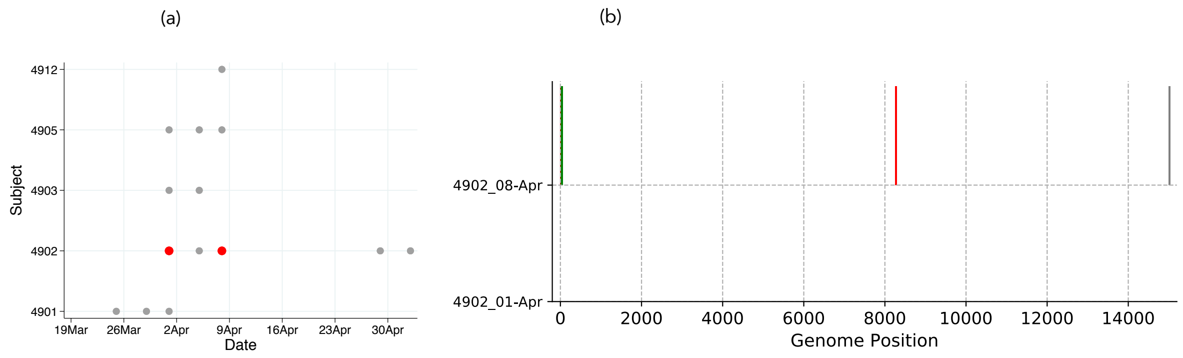


**S19 Figure.** Patterns of RSVA infection in household 49 (HH49) in which 5/12 members got infected. Sequencing was successful for only one member while the other four infected members all their positive samples failed sequencing. The legend details for *Panel A-D* are same as S3 Figure.

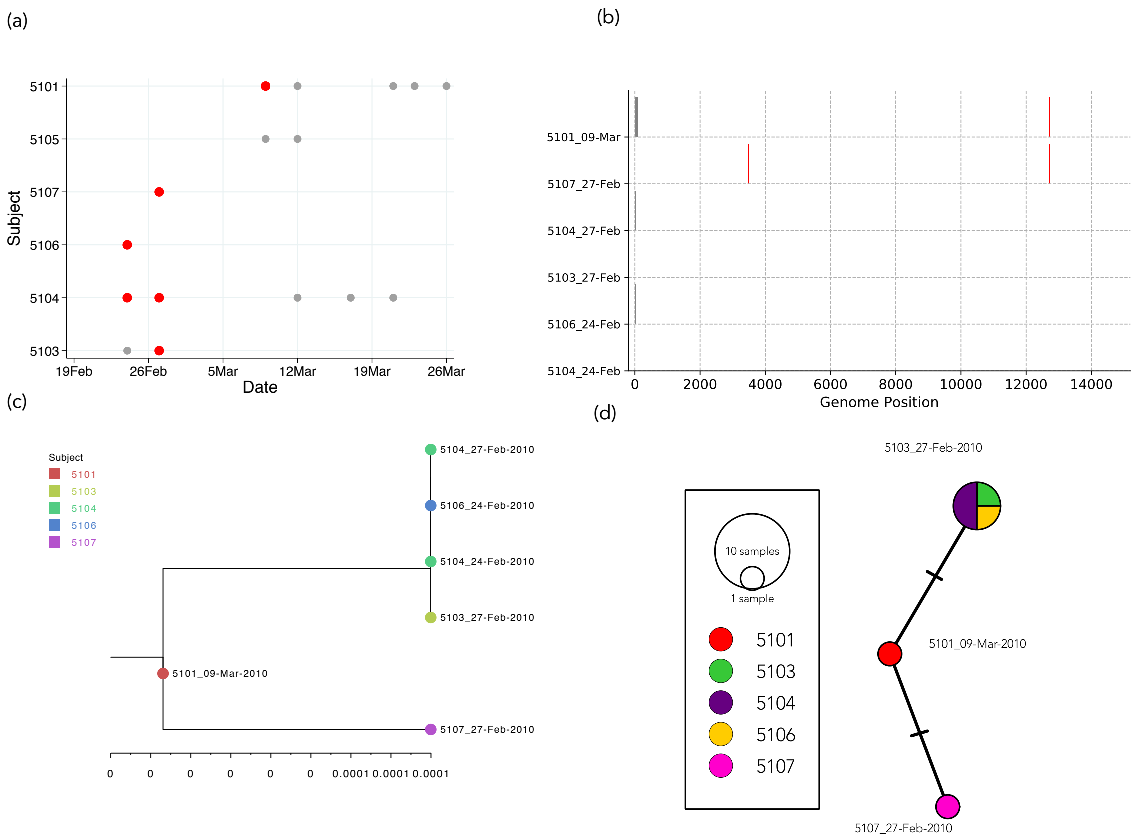


**S20 Figure.** Patterns of RSVA infection in household 51 (HH51) in which 6/15 members got infected. For one of the infected members all two positive samples failed sequencing. The legend details for *Panel A-D* are same as S3 Figure.

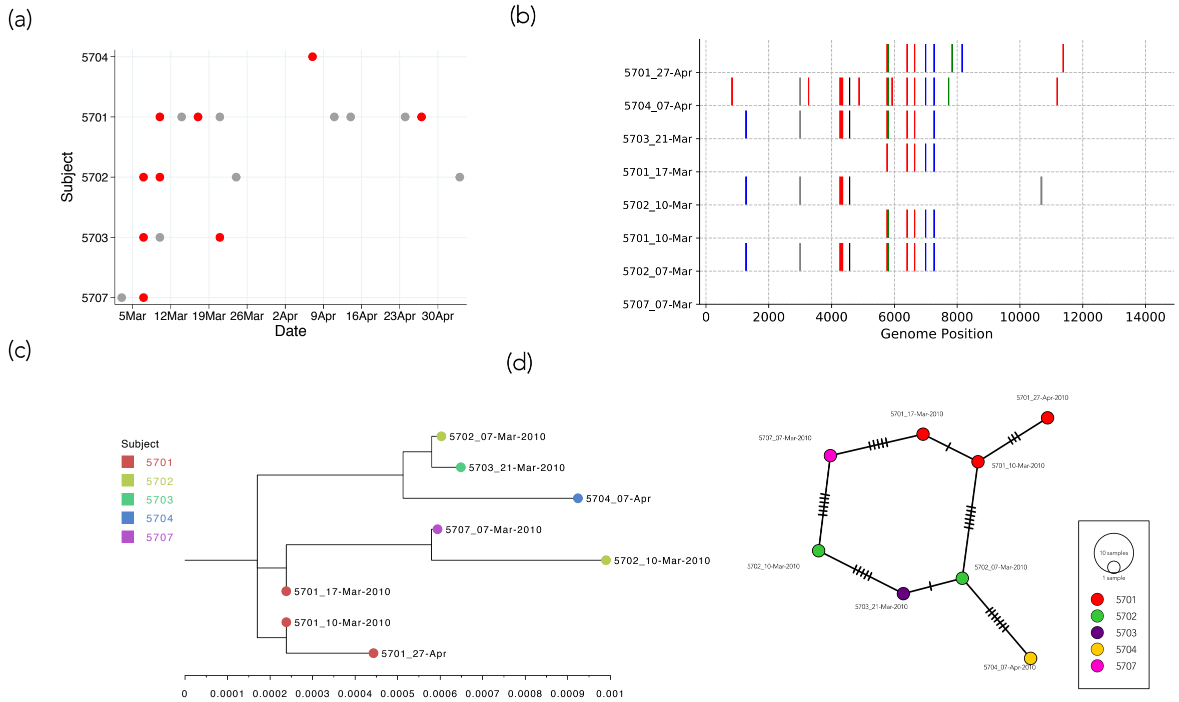


**S21 Figure.** Patterns of RSVA infection in household 57 (HH57) in which 5/16 members got infected. The legend details for *Panel A-D* are same as S3 Figure.
